# Amyloid-induced mitochondrial network disruption in neurons monitored by STED super-resolution imaging

**DOI:** 10.3389/fcell.2025.1610204

**Published:** 2025-06-10

**Authors:** Iuliia Golovynska, Qinglin Chen, Yurii V. Stepanov, Danying Lin, Junle Qu

**Affiliations:** ^1^ State Key Laboratory of Radio Frequency Heterogeneous Integration (Shenzhen University), College of Physics and Optoelectronic Engineering and Key Laboratory of Optoelectronic Devices and Systems of Ministry of Education and Guangdong Province, Shenzhen University, Shenzhen, China; ^2^ Laboratory of Molecular and Cellular Mechanisms of Metastasis, R.E. Kavetsky Institute of Experimental Pathology, Oncology and Radiobiology, NAS of Ukraine, Kyiv, Ukraine

**Keywords:** Alzheimer’s disease, amyloid-beta, neuron mitochondria, mitochondrial network analysis, STED imaging

## Abstract

**Introduction:**

Disruptions in mitochondrial metabolism are accompanied by morphological changes in mitochondrial network caused by amyloid-beta (Aβ). In the study, mitochondrial network analysis is performed using stimulated emission depletion (STED) super-resolution fluorescence microscopy to examine alterations in neurons exposed to Aβ *in vitro*.

**Methods:**

A detailed analysis of mitochondrial network in healthy neurons and those exposed to Aβ is performed using STED compared to conventional laser-scanning confocal fluorescence microscopy. The functional analysis is applied to mitochondrial volume, surface area, branch length, diameter, junctions, and endpoints. Neurons incubated with or without Aβ were also stained with fluorescent mitochondrial function indicators.

**Results:**

In neurons exposed to Aβ, the number of mitochondria increases by 2.6 times, while their total volume decreases by 2.2 times. As a result, the volume and surface area per mitochondrion decrease by 6-fold and 4-fold, respectively. Increases in sphericity, branch diameter, and donut-like structures are observed. The total mitochondrial length is 3.7-fold reduced, while the number of branches is 2.5-fold decreased, and the branch count is 7.5-fold reduced. Additional measurements reveal decreased mitochondrial membrane potential, increased reactive oxygen species generation, and reduced cell viability. This may indicate that Aβ exposure causes significant oxidative stress, mitochondrial integrity loss, and ultimately neuronal death.

**Conclusion:**

Aβ induces mitochondrial fragmentation, thickening, increased sphericity, and deformation of mitochondrial matrix in neurons. The results provide insights into the impact of Aβ on neurons and show the aptitude of the high-resolution STED microscopy diagnostic tool for neurodegenerative diseases.

## 1 Introduction

Alzheimer’s disease (AD) is a complex neurodegenerative disorder characterized by cognitive decline and dementia. Pathologically, the disease is strongly associated with neuron loss due to mitochondrial dysfunction and oxidative stress. Amyloid-beta (Aβ) accumulation impairs mitochondrial function, resulting in harmful effects on neuronal cells. Simultaneously, the disruptions in mitochondrial function are accompanied by alterations in the mitochondrial network (Aran and Singh, 2023).

Mitochondria are essential organelles in cells, responsible for generating adenosine triphosphate (ATP) through oxidative phosphorylation, which serves as the cell’s main energy source. They also regulate cellular metabolism, calcium homeostasis, inflammation, and immunity (Eisner et al., 2018). Mitochondrial dynamics and network morphology fluctuate under different cellular conditions, correlating with energy storage (Bleck et al., 2018), and play key roles in organelle quality control (Rafelski, 2013). Mitochondria constantly change shape by the combined actions of fusion, fission, and movement along microtubule tracks. The balance between rates of mitochondrial biogenesis (formation of fresh mitochondrial material) and mitophagy (mitochondrial autophagy that removes damaged mitochondria) determines mitochondrial branch length and network formation (Rafelski, 2013). The fusion helps mitigate stress by providing mitochondrial DNA complementation, mixing healthy and impaired mitochondrial DNA (Rong et al., 2021). In this way, damaged sections can be repaired within the network (Rafelski, 2013). On the other hand, the fission creates new mitochondria and isolates the fragment with damaged DNA, facilitating apoptosis at high levels of oxidative stress (Rong et al., 2021). During apoptosis, mitochondria often become fragmented and swollen, with disrupted metabolism (Van Der Bliek et al., 2013; Eisner et al., 2018).

The disruption in the coherence between mitochondrial fusion and fission in neurons leads to neurological disorders (Mishra and Chan, 2014). Mitochondrial fission is regulated by Dynamin-related protein 1 (DLP1), triggering its fragmentation (Randazzo et al., 2013). In AD pathology, increased mitochondrial fission is linked to abnormal interactions between oligomeric Aβ, DLP1, and hyperphosphorylated tau protein (Manczak et al., 2011; Kandimalla et al., 2018; Reiss et al., 2022). Mitochondria fusion, however, is controlled by specific dynamin family proteins: mitofusin-1 and mitofusin-2 binding to the outer mitochondrial membrane (Filadi et al., 2018; Han et al., 2020) and the optic atrophy type 1 protein binding to the inner membrane (Szabo et al., 2018; Ge et al., 2020). Imbalances in these proteins result in hyper-fragmented mitochondrial networks (Ge et al., 2020; Zacharioudakis et al., 2022), metabolic dysfunction, and the development of neurodegenerative diseases (Van Der Bliek et al., 2013; Filadi et al., 2018).

Neurons exposed to Aβ exhibit mitochondrial dysfunction (Reddy and Beal, 2005; Moreira et al., 2007), reduced membrane potential, increased reactive oxygen species (ROS) generation, oxygen-glucose deprivation, and altered mitochondrial network morphology (Barsoum et al., 2006; Sirk et al., 2007). Aβ inhibits neuronal differentiation (Kim et al., 2022) and promotes mitochondrial dysfunction (Li et al., 2023) and fragmentation (Barsoum et al., 2006; Rui and Zheng, 2016), which occur through H_2_O_2_-dependent Src kinase activation. These processes involve diverse cellular events, such as Ca^2+^ receptor activation and increased intracellular Ca^2+^ concentration (Manczak and Reddy, 2012; Rui and Zheng, 2016; Mota et al., 2023). However, studies on mitochondrial network morphology alterations after Aβ exposure *in vivo* and *in vitro* mainly focus on its fragmentation (Sirk et al., 2007; Randazzo et al., 2013; Rui and Zheng, 2016; Reiss et al., 2022) without analyzing other key aspects like mitochondrial volume, diameter, sphericity, or junctions. Additionally, mitochondrial dynamics during cell division (mitosis) are often overlooked (Taguchi et al., 2007; Carlton et al., 2020; Pangou and Sumara, 2021), even though mitochondrial dynamics during pathological processes and mitotic progression may appear similar. Thus, objective comprehensive quantification of mitochondrial network alterations is essential for understanding mitochondrial health. Stimulated emission depletion super-resolution fluorescence microscopy (STED-FM or shortened to STED), a type of super-resolution imaging using spatially structured excitation, has become a powerful tool in biological research, offering new opportunities for three-dimensional (3D) visualizing and analyzing mitochondrial structures (Ishigaki et al., 2016; Stephan et al., 2019; Yang et al., 2020; Wang et al., 2021; Ren et al., 2024; Hirtl et al., 2025). Yet, this powerful technique was not used to measure changes in the mitochondrial network after Aβ exposure.

In this study, we present a detailed analysis of mitochondrial network in neurons exposed to Aβ, using STED and comparing it to conventional laser-scanning confocal fluorescence microscopy (CFM). The functional analysis and optimized thresholding are applied to measure changes in mitochondrial volume, surface area, branch length, diameter, junctions, and endpoints. Additional measurements reveal changes in mitochondrial membrane potential (MtMP), ROS concentration, and cell viability after Aβ exposure.

## 2 Methods

### 2.1 Neuron culture and cultivation

HT-22 mouse hippocampal neuronal cells (Procell Life Science and Technology, China) were used immediately after purchase. The cells were cultured in a complete medium for hippocampal neurons (Eagle’s minimum essential medium EMEM/F12 with 15% FBS and 1% penicillin-streptomycin solution from Procell Life Science and Technology, China) at 37°C in a 95% humidified atmosphere with 5% CO_2_ in a standard incubator. For fluorescence imaging during long-term live cell experiments, we used an ibidi Stage Top Incubator (ibidi, Germany) with temperature control (37°C), 5% CO_2_ maintenance, and 90% humidity.

### 2.2 Oligomeric β-amyloid (1–42) preparation and use

Neurons were treated with soluble oligomeric forms of Aβ1-42, recognized as the most neurotoxic Aβ species (Walsh et al., 2002; Benilova et al., 2012). Aβ1-42 (Thermo Fisher Scientific, United States) was oligomerized following an established protocol (Stine et al., 2010), as detailed in our prior studies (Stepanov et al., 2022; Golovynska et al., 2024). A concentration of 10 μM oligomeric Aβ1-42 was used to induce observable cellular changes in a short timeframe during experimental modeling. While Aβ1-42 naturally occurs in the brain at picomolar concentrations (Kass et al., 2022) and affects neurons over the years in AD, such low concentrations do not significantly affect neuron viability *in vitro* over several days. Independent studies (Dahlgren et al., 2002; Stepanov et al., 2022) have shown that 10 µM is a suitable concentration for neuron-culture experiments (Xie et al., 2023; Uzoechi et al., 2024). The cultivation of neurons with Aβ during 24 h was chosen because no significant difference was observed after either 6 or 12 h, while a low cell viability of ∼50% was assessed after 48 h, which complicates cell staining and mitochondria quantification.

### 2.3 Mitochondria STED microscopy imaging and 3D mitochondrial analysis

Neurons were placed into eight 35-mm glass-bottom dishes and incubated with or without Aβ for 24 h (four dishes for each group). After cultivation, the used medium was removed, and neurons were washed thoroughly to remove a Aβ-containing medium (a further escalation of Aβ-induced alterations were considered minimal). Immediately after that the cells were stained with 1 μg/ml PK Mito Orange (PKMO) in a medium for 15 min at 37°C (Liu et al., 2022). The mitochondrial inner membrane fluorescent PKMO marker, with *λ*_*exc*_ = 590 nm/*λ*_*em*_ = 610 nm (Nanjing Genvivo Biotech, China), was used for labeling neurons to detect live mitochondria and further reconstruct mitochondrial network. This marker has a reduced phototoxicity for time-lapse imaging, compatible with commercial STED nanoscopes. Following staining, neurons were washed thoroughly and placed in a phenol red-free medium to minimize optical absorption and heating during laser excitation.

FM imaging was performed using an Abberior STEDYCON microscope system (Abberior Instruments, Germany) built on an Olympus IX83 inverted fluorescence microscope equipped with a UPlanXAPO 100X/NA1.45 objective (Olympus, Japan). Operating in CFM modality, laser excitation of 561 nm was used, resulting in a lateral resolution of 193 nm. For STED imaging, an excitation laser of 561 nm and a depletion laser of 775 nm were used. Planar images in *x-* and *y*-axes were combined with *z*-stack acquisitions. For the STED lateral resolution, we performed resolution measurement on Hela cells, using the same probe. The results gave an average resolution of 85 nm (Supplementary Figure S1). The lateral sampling rate was set as 25 nm, fulfilled the Nyquist sampling theorem, the pixel dwell time was set as 10 μs with a three line average, the scanning time for an image of 12.8 × 12.8 μm^2^ (512 × 512 pixel^2^) was ∼8 s. For 3D cell imaging, the z-size was ∼18 μm and the optimal *z*-spacing was 100 nm. Such parameters result in acquiring a whole z-stack STED scan during ∼30 min. Other parameters like laser power, detector gating, and gain were optimized to reduce background noise, prevent signal saturation, and minimize photobleaching.

**FIGURE 1:**
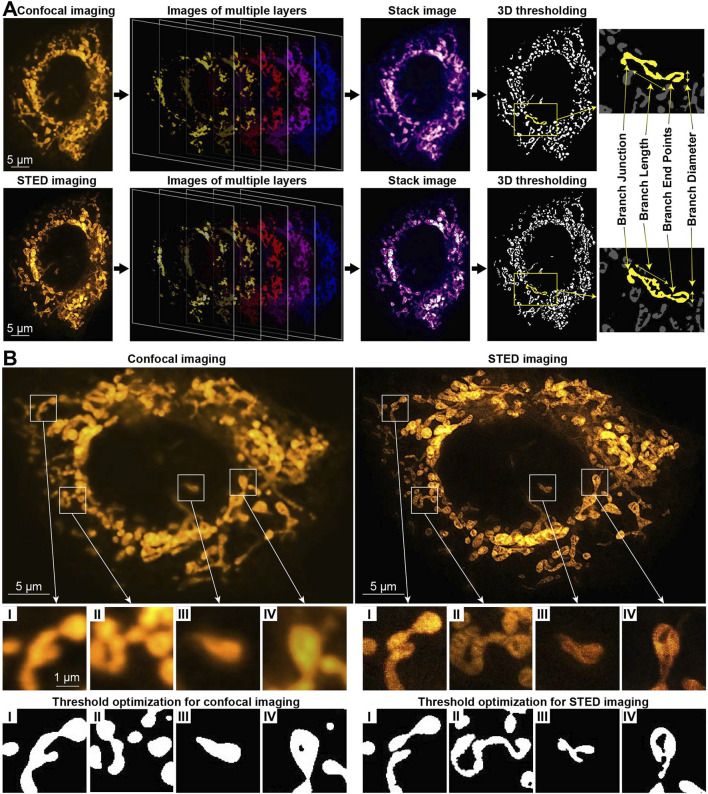
3D mitochondrial analysis in neurons using fluorescence confocal and STED microscopy. **(A)** Representative image showing mitochondria labeled with PKMO marker, emitting yellow light. It includes time-lapse z-slices, stack image projection, 3D reconstruction, and quantification of mitochondrial network morphology in a healthy neuron. **(B)** Comparative analysis of 2D confocal and STED images with threshold normalization for a neuron treated with Aβ.

The images were processed with ImageJ software, with raw data displayed unless specified otherwise. A full mitochondrial reconstruction was created by integrating stacked slices with ImageJ/Fiji. Z-stakes captured detailed mitochondrial networks, displayed in pseudo-color images (Figure 1A). Using Mitochondrial Analyzer 3D plugin (Valente et al., 2017; Hemel et al., 2021), we quantified mitochondrial count, volume, surface area, length, diameter, sphericity, number of branches, junctions, and endpoints. Thresholding and contrast optimization were automatically adjusted (Chaudhry et al., 2020), ensuring precise network delineation and distinguishing tightly packed mitochondrial components.

### 2.4 Mitochondrial membrane potential, ROS, and viability assays using epifluorescence microscopy

Neurons were placed into 35-mm glass-bottom dishes and incubated with mitochondrial function indicators. An incubation medium contained Image-iT™ TMRM (red-fluorescent, 588/613 nm, diluted at 1 μg/mL) to measure MtMP, or MitoSOX™ (green-fluorescent, 488/510 nm, 2 μg/mL) to detect mitochondrial ROS (Thermo Fisher Scientific, United States). To assess cell viability and necrosis, neurons were stained with Calcein AM (green-fluorescent, 494/517 nm, 1 μg/mL) for live cells and propidium iodide (PI, red-fluorescent, 535/617 nm, 1 μg/mL) for dead cells. After probe incubation, the cells were washed and placed in a phenol red-free medium for optical imaging. Fluorescence images were captured using a Nikon Eclipse Ti-U microscope with a 40X objective (for MtMP and ROS) and a 20X objective (for cell viability) and equipped with a Nikon Digital camera. The images were analyzed with NIS-Elements Viewer 4.50 software to evaluate fluorescence intensity. The Nikon dual-bandpass excitation and emission filters were used for fluorescence imaging (Nikon, Tokyo, Japan).

### 2.5 Statistical analysis

Statistical comparison between the groups of Aβ-treated neurons and control untreated neurons was performed using a Student’s t-test, *N* = 20. Biological replicates were performed on 20 independent cells in four dishes for each group; technical replicates were performed 2–3 times per cell, and there were no observed changes between repetitions. The data were normally distributed. Results are presented as the mean ± standard deviation (M ± SD). Differences were considered significant at *p* < 0.05.

## 3 Results

### 3.1 Comparison of neuron mitochondria using confocal versus STED imaging

Combined CFM and STED imaging was performed on neurons stained with the mitochondrial dye PKMO. For 3D mitochondrial analysis, a stack of confocal images along the *z*-axis was captured and processed. This was followed by 3D functional analysis and threshold optimization to quantify mitochondrial network morphology. The quantification includes parameters, such as total count, volume, surface area, diameter, length, sphericity, number of branches, junctions, and endpoints. The total count represents all identified mitochondrial matrix objects and is interconnected with the total volume and surface area of each cell. The average volume and surface area per mitochondrion were also calculated by dividing these values by the total count (Valente et al., 2017). These parameters are crucial for providing more precise insights into a mitochondrial network in each case. Moreover, no single parameter, when considered alone, can definitely distinguish between physiological and pathological fission. Figure 1A represents the CFM and STED imaging comparison for 2D raw images, 3D reconstruction procedure, and structural components of mitochondria from a neuron.

In healthy neurons with an intact mitochondrial network, the CFM and STED images appear similar (Supplementary Figure S2), allowing mitochondrial network quantification of total count, volume, and surface area without substantial loss of details using either imaging modality (Supplementary Figure S3). At the same time, the parameters of branch count, length, and junctions are significantly higher when being identified by STED (Supplementary Figure S3). Therefore, CFM analysis becomes inadequate for neurons with a more segregated mitochondrial network, such as those cultured for 24 h in the presence of Aβ, which disrupts the network morphology. For an Aβ-treated neuron, Figure 1B and Supplementary Figure S3 illustrate the comparison between 2D CFM/STED raw images and black-and-white ones with threshold optimization applied. Due to their higher spatial resolution, the STED images provide significantly clearer patterns after threshold optimization, compared to CFM. For example, in the STED image in Figure 1B I, two long mitochondria are distinguishable, whereas they appear as a single structure in the CFM image in Figure 1B I. Similarly, in Figure 1B II, a long mitochondrion visible in the STED image appears fragmented in the CFM image in Figure 1B II. Figure 1B III, IV further highlight the lack of clarity in the CFM images, leading to potentially inaccurate quantification. Thereby, for Aβ-treated neurons, the quantification gives the parameters of branch count, length, junctions, and endpoints significantly higher by STED (Supplementary Figure S3).

Thus, for precise quantification of mitochondrial network, STED imaging proves its superiority compared to CFM due to enhanced spatial resolution. A major limitation of STED microscopy is the relatively high donut light beam intensity required for the depletion process, which can cause photobleaching and phototoxicity. Although we chose anti-photobleaching fluorescent probe PKMO for staining, a strong light exposure may still pose photodamage to the cells. The exploited STEDYCON system has excellent lateral resolution of about 85 nm in our experiments, while its axial resolution is still nearly 0.6 μm, like the conventional confocal microscope. In addition, the high lateral resolution of STED imaging requires a high sampling rate, which means longer acquisition time for each imaging plane, when living cells are moving objects. Thus, CFM is a better choice for quick 3D examination. For the aforementioned reasons, the STED results are further deliberated for Aβ-induced alterations in neuronal mitochondria.

### 3.2 Aβ-induced changes in mitochondrial morphology observed using STED imaging

**FIGURE 2:**
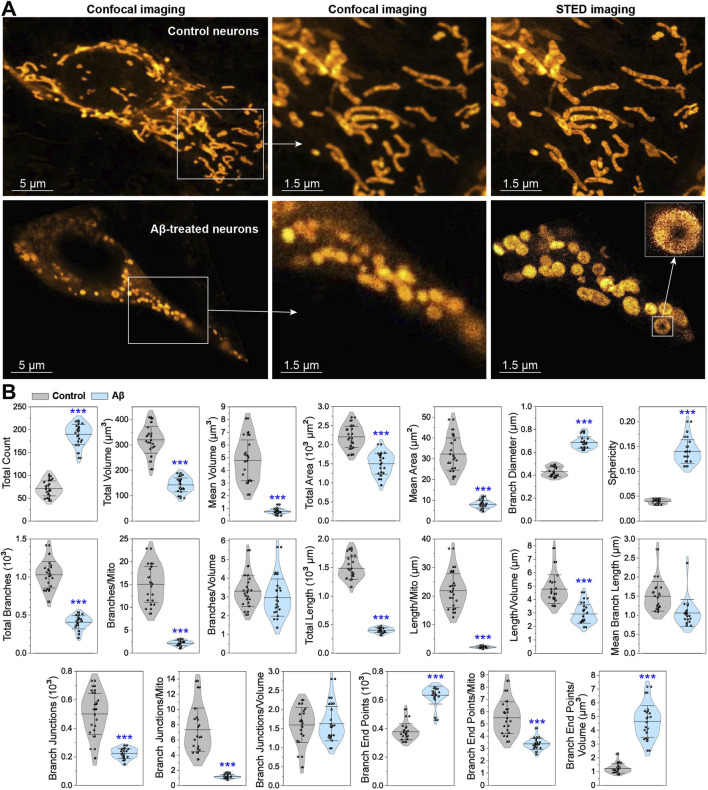
Quantification of mitochondrial disruptions in neurons under Aβ overload. **(A)** Representative confocal and STED images of control and Aβ-treated neurons stained with PKMO marker. **(B)** Graphs displaying the quantification of mitochondrial parameters, including count, volume, surface area, branches, length, junctions, end points, sphericity, and diameter. Data are presented as M ± SD, with ***p < 0.001 indicating a statistically significant difference (Student’s t-test).

The STED analysis of mitochondrial network structure, connectivity, and formation relies on threshold-based measurements. Representative STED images in Figure 2A illustrate the fragmentation of the mitochondrial network in Aβ-treated neurons compared to healthy neurons, and the corresponding quantified parameters are presented in Figure 2B. After exposure to Aβ, the total count of mitochondrial objects increases from 72 to 190 (by 2.6 times). Conversely, the total volume decreases from 320 to 143 μm^3^ (by 2.2 times), and the average volume per mitochondrion decreases drastically from 4.8 to 0.8 μm^3^ (6-fold). The total surface area is reduced from 2,206 to 1,501 μm^2^ (by 1.5 times), while the average surface area drops from 32 to 8 μm^2^ (4-fold). This increase in the number of mitochondrial objects, combined with the reduction in their volume and surface area, indicates significant pathological disruptions, where mitochondria become fragmented and shrunken. This can be concluded because the fragmentation during physiological restructuring does not cause the loss of mitochondrial volume (Taguchi et al., 2007).

The branch diameter and sphericity are the key parameters indicating abnormalities in the mitochondrial network. Cells entering apoptosis are often characterized by mitochondrial fragmentation, thickening, rounding, and deformation (Van Der Bliek et al., 2013). While fragmentation can occur during normal physiological processes (Taguchi et al., 2007; Carlton et al., 2020; Pangou and Sumara, 2021), mitochondrial matrix swelling is predominantly associated with pathological conditions (Sun et al., 2007; Shamseldin et al., 2021). Mitochondrial swelling is usually irreversible, with swollen and fragmented mitochondria being eliminated by lysosomes (Ryan et al., 2020; Xian and Liou, 2020). Increased presence of swollen mitochondria ultimately leads to cell apoptosis and necrosis (Sun et al., 2007; Twig et al., 2008; Rafelski, 2013). Abnormal fusion events lead to the formation of donut-shaped (toroidal) mitochondria, which is a unique type of mitochondrial fragmentation. The donut formation is triggered by the opening of the potassium channels onto the mitochondrial membrane, leading to high osmotic pressure inside mitochondria. This event also causes mitochondrial swelling, bending with partial detachment from the cytoskeleton, and eventual formation of a donut (Liu and Hajnóczky, 2011). The representative images and sphericity graph in Figure 2 illustrate the characteristic shape of these mitochondria with markedly higher sphericity. The donut formation is also observed in Aβ-treated neurons, with approximately six donuts per cell image, alongside an increase in branch diameter from 0.43 to 0.69 μm (by 60%). These findings suggest that Aβ disrupts ion flow into mitochondria, leading to swelling and fragmentation.

The branching of mitochondrial network is quantified by measuring the total branch count, the average number of branches per mitochondrion, and the branches per mitochondrion volume for each analyzed cell (Figure 2B). The total branch count decreases significantly from 1,029 to 405 (by 2.5 times), and the average number of branches per mitochondrion drops from 15 to 2 (by 7.5 times). However, the number of branches per volume remains unchanged, indicating substantial degradation of mitochondria.

Combining mitochondrial length data with other parameters provides additional insights into the extent of network fragmentation. Mitochondrial length is measured as parameters of the total length per cell and the average length per mitochondrion, volume, or branch. As shown in Figure 2, Aβ-treated neurons exhibit a significant reduction in mitochondrial length: the total length decreases from 1,489 to 403 μm (by 3.7 times), the average length per mitochondrion reduces from 22 to 2 μm (11-fold), the length per volume decreases from 4.8 to 2.9 μm (by 40%), and the average branch length decreases from 1.5 to 1.1 μm (by 27%). Considering the increase in the total number of mitochondria, their diameter and sphericity, along with the reduced length, these changes in Aβ-treated neurons point to severe pathological fragmentation.

Mitochondrial branch junctions reflect the connectivity of the network, changed due to active remodeling processes like fusion, fission, shape transitions, transport, or tethering along the cytoskeleton (Lackner, 2014). The total junction count and average number of junctions per mitochondrion or per volume quantify these changes. As shown in Figure 2B, the total junction count decreases sharply from 500 to 223 μm (by 2.2 times), and the number of junctions per mitochondrion drops from 7.3 to 1.2 μm (6-fold). However, the number of junctions per volume remains static because the branch count per volume is unchanged. The reduction in the branch junction count suggests that mitochondrial fusion is almost or completely absent, and only fission occurs.

The endpoints of mitochondrial branches are similarly quantified, including the total number of branch endpoints per cell and the average number of endpoints per mitochondrion or per volume. In Figure 2B, the total endpoint count increases from 378 to 634 μm (by 68%), while the endpoint count per volume increases from 1.2 to 4.6 μm (by 283%). However, the endpoint count per mitochondrion decreases from 5.5 to 3.4 μm (by 38%). These findings further confirm the predominance of mitochondrial fission.

In summary, we assessed all measurable and calculable parameters from the STED images of mitochondrial matrix in healthy neurons and those after Aβ exposure and analyzed the correlation between them. The results suggest that Aβ induces significant pathological fragmentation, thickening, rounding, and deformation of mitochondrial matrix in neurons.

### 3.3 Mitochondrial metabolic changes in neurons exposed to Aβ

The structural abnormalities of the mitochondrial network in Aβ-treated neurons are accompanied by disruptions in metabolism. Mitochondrial fusion relies on a stable MtMP (Legros et al., 2002; Meeusen et al., 2004). However, swollen regions of the network, which lose their ability to function properly and generate MtMP, become isolated and are targeted for mitophagy (Sun et al., 2007; Twig et al., 2008; Rafelski, 2013). The fragmentation of mitochondrial network is also associated with reduced respiration, increased oxidative phosphorylation, and elevated generation of mitochondrial ROS (Nagdas and Kashatus, 2017). Under moderate oxidative stress, mitophagy helps clear defective parts of mitochondria, lowering ROS concentrations and improving cell survival. In contrast, severe oxidative stress triggers excessive mitochondrial fission and dysfunction, leading to a heightened ROS production, loss of mitochondrial integrity, and ultimately apoptotic cell death (Ježek et al., 2018). Aβ accumulation is widely recognized to induce severe oxidative stress during the progression of neurodegenerative diseases, particularly AD (Cheignon et al., 2018).

**FIGURE 3:**
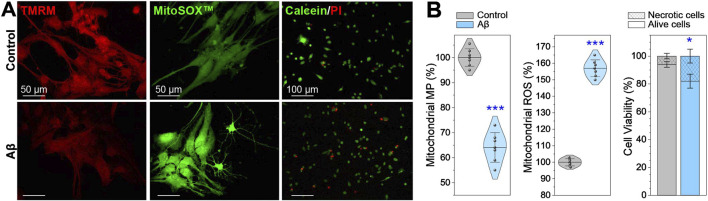
Mitochondrial membrane potential (MtMP), reactive oxygen species (ROS) production, and cell viability in Aβ-treated neurons compared to healthy control. **(A)** Representative fluorescence images and **(B)** charts showing neurons stained with Image-iT™ TMRM for MtMP, MitoSOX™ for mitochondrial ROS, and PI/Calceine for cell viability. Data are presented as M ± SD, with *p < 0.05 and ***p < 0.001 indicating statistically significant differences (Student’s t-test).

The fluorescent images and data in Figure 3 reveal a 37% reduction in MtMP in neurons treated with Aβ for 24 h. Concurrently, ROS production increases by 57%, while cell viability decreases by 12%. The correlation between changes in MtMP, ROS concentration, and the mitochondrial network morphology parameters in Aβ-treated neurons is determined by calculating the statistical Pearson correlation coefficient (more details are in Supplementary Materials). An inverse correlation is found between the number of mitochondria (total count) and the decrease in MtMP, meanwhile, a direct correlation is noticed between the ROS concentration and the number of mitochondrial objects. The reduction in the total branch length, total branch length/mito, branch junctions, and branch junctions/mito has a direct correlation with the decreased MtMP and an inverse correlation with the increased ROS. Moreover, the decreased MtMP and increased ROS concentration strongly correlate.

These findings suggest that the decreasing MtMP and increasing ROS concentration after Aβ exposure may have a negative impact on mitochondria, leading to their fragmentation and reduction in branch length and connectivity. These events are related to oxidative damage, as ROS are highly reactive molecules that can damage various cellular components, including lipids, proteins, and mitochondrial DNA. The damage to mitochondrial DNA disrupts the processes of fission and fusion of mitochondria, leading to their fragmentation, thickening, spheroidization, and eventual neuron death.

## 4 Conclusion

A detailed analysis of mitochondrial network in healthy neurons and those exposed to Aβ is performed using STED, comparing it to conventional CFM. Planar images and mitochondrial network reconstruction were analyzed to provide high-quality, clear interpretations of mitochondrial disruptions. Functional analyses and optimized thresholds were applied to quantify parameters such as mitochondrial volume, area, branch length, diameter, junctions, and endpoints. The comparative analysis shows that, for precise quantification of mitochondrial network, STED imaging proves superior to CFM due to its enhanced spatial resolution. Thus, STED results were further used to study alterations of the mitochondrial network in neurons exposed to Aβ.

STED microscopy showed that the number of mitochondria increased by 2.6 times, while their total volume decreased by 2.2 times. As a result, the volume and surface area per mitochondrion decreased by 6-fold and 4-fold, respectively. Significant changes were also observed, including increased sphericity, the formation of donut-like structures, and a thickening of branch diameters. Mitochondrial length decreased by 3.7-fold. The number of branches dropped by 2.5 times, while the average branch number per mitochondrion reduced by 7.5-fold. These findings suggest that Aβ may trigger severe pathological effects in neurons, including fragmentation, thickening, spheroidization, and deformation of mitochondrial matrix. Additional measurements revealed decreased MtMP, increased ROS generation, and reduced cell viability, indicating that Aβ exposure causes significant oxidative stress, mitochondrial integrity loss, and ultimately neuronal death.

We analyzed all measurable parameters from the STED images, and their correlative analysis allowed us to deliver a comprehensive and accurate representation of mitochondrial network fragmentation after Aβ exposure. This holistic microscopy approach for analyzing cell samples may offer potential for early detection of pathological changes in cellular physiology.

## Data Availability

The raw data supporting the conclusions of this article will be made available by the authors, without undue reservation.
