# Supplementary material for: Amyloid-induced mitochondrial network disruption in neurons monitored by STED super-resolution imaging
Source: Front Cell Dev Biol. 2025 Jun 10;13:1610204. doi: 10.3389/fcell.2025.1610204 (PMC12185474; doi:10.3389/fcell.2025.1610204)
Supplement: Supplementary file 1 [file DataSheet1.pdf]

Supplementary Materials

Amyloid-induced mitochondrial network disruption in neurons monitored by STED super-resolution imaging

Iuliia Golovynska [a,#], Qinglin Chen [a,#], Yurii V. Stepanov [b], Danying Lin [a,*], Junle Qu [a,*]

[a] State Key Laboratory of Radio Frequency Heterogeneous Integration (Shenzhen University), College of Physics and Optoelectronic Engineering & Key Laboratory of Optoelectronic Devices and Systems of Ministry of Education and Guangdong Province, Shenzhen University, Shenzhen 518060, P. R. China

[b] Laboratory of Molecular and Cellular Mechanisms of Metastasis, R.E. Kavetsky Institute of Experimental Pathology, Oncology and Radiobiology, NAS of Ukraine, Kyiv 03022, Ukraine

[#] These authors contributed equally to this work and share first authorship

[*] **Correspondence:**

[*] Corresponding authors: Danying Lin (dylin@szu.edu.cn) and Junle Qu (jlqu@szu.edu.cn)

Methods

Spatial resolution of STED imaging

To showcase the resolution of STED imaging, we conducted STED imaging on Hela cells, using PKMO fluorescent probe. To measure the resolution of STED images, we applied single-frame rFRC method [1]. The action was done by ImageJ plugin PANELJ-Single-frame rFRC mapping (1/7 threshold, beta), 50 STED images were involved. The results in Figure S1 suggest the average resolution of ~ 85 nm.

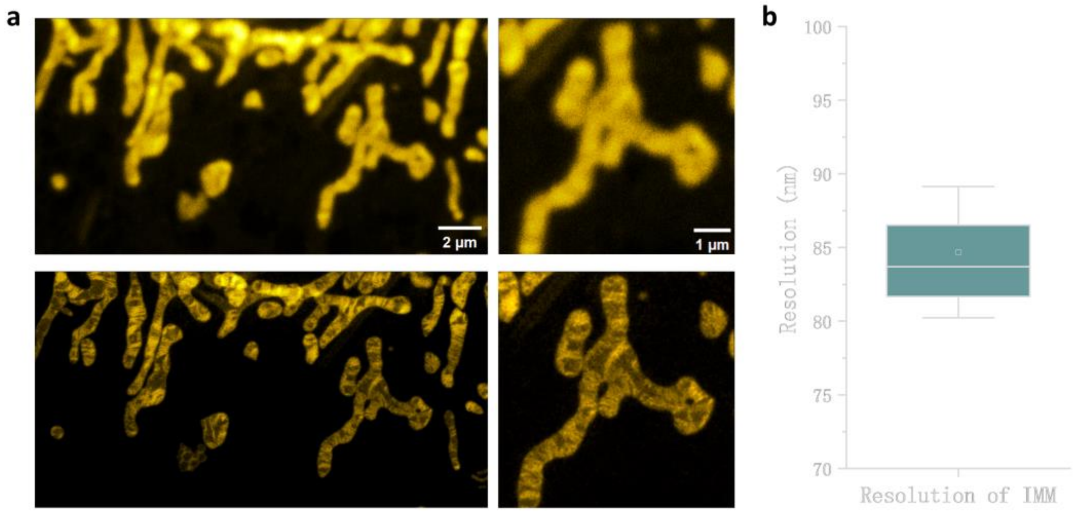

Figure S1. STED imaging results and corresponding resolution statistics. **(a)** Images of confocal and STED imaging modalities, showing the overall and magnified FOV. **(b)** Statistical resolution measurements of STED images.

Supplementary References

1. Zhao, W. S., Huang, X. S., Yang, J. Y. et al. Quantitatively mapping local quality of super-resolution microscopy by rolling Fourier ring correlation. *Light Sci. Appl.* **12**, 298 (2023).

Results

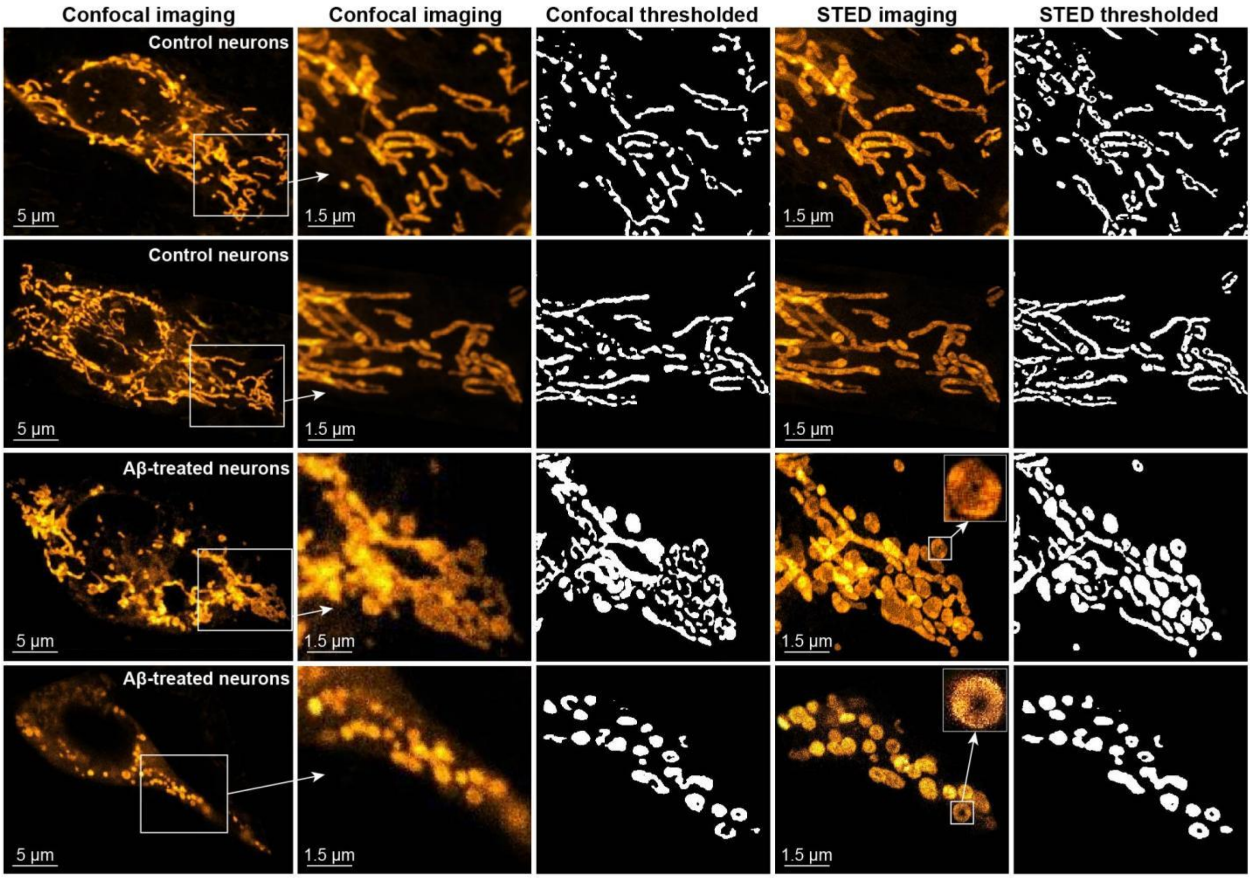

Figure S2. Comparative quantification of mitochondrial destruction in neurons after Aβ load using confocal fluorescence microscopy (CFM) and stimulated emission depletion super-resolution fluorescence microscopy (STED). Representative images of control and Aβ-treated neurons stained with PKMO. The functional analysis and optimized thresholding are applied to measure changes in mitochondrial volume, surface area, branch length, diameter, junctions, and endpoints.

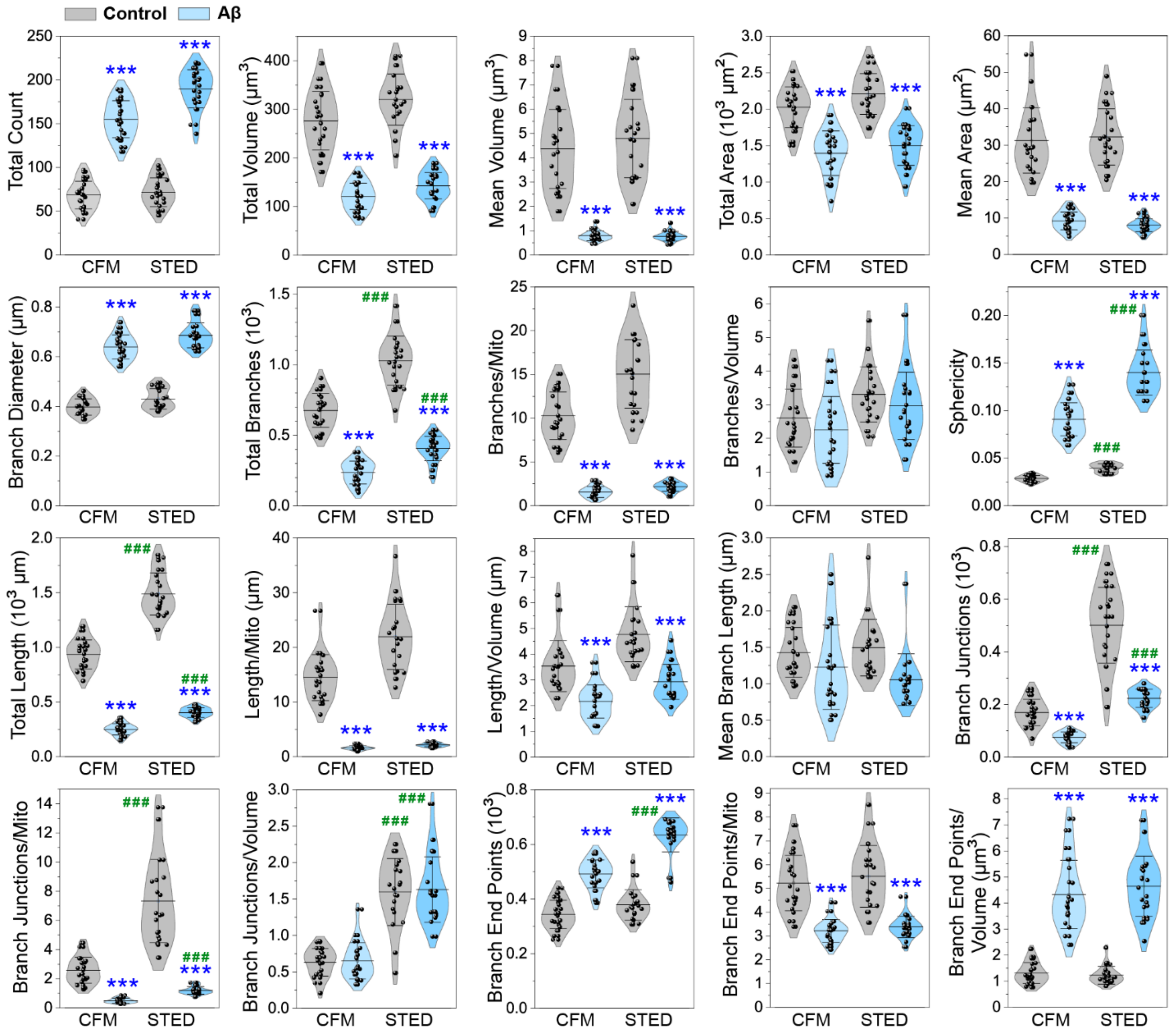

Figure S3. Comparative quantification of mitochondrial disruptions in neurons after Aβ load using CFM and STED. Violin plots with data distribution displaying the mitochondrial parameters, including count, volume, surface area, branches, length, junctions, end points, sphericity, and diameter. Data are presented as M ± SD, with ***p < 0.001 indicating a statistically significant difference compared to the control and $^{###}p$ < 0.001 indicating a statistically significant difference between the same experimental group measured using CFM and STED (Student's *t*-test).

Pearson correlation coefficient

The first criterion is the normality of the distribution: if the distribution of values is normal, then a decision is made to select the linear correlation coefficient r-Pearson; if the distribution of values is not normal, then a decision is made to select the rank correlation coefficient *r*-Spearman. In our case, the distribution is normal, so the calculation of the Pearson correlation coefficient (r) was done:

$$r = \Sigma[(x_i - \bar{x})(y_i - \bar{y})] / \sqrt{[\Sigma(x_i - \bar{x})^2 \times \Sigma(y_i - \bar{y})^2]}$$

where x_i and y_i are the values of the i-th element of variable X and Y, respectively; and $\bar{x}$ and $\bar{y}$ are the average value of variable X and Y, respectively.

The correlation between changes in MtMP, ROS concentration, and mitochondrial network morphology (total count, total branch length, total branch length/mito, branch junctions, and branch junctions/mito) was determined in Aβ-treated neurons. The statistical method of calculating the Pearson correlation coefficient (r) was used for the correlational analysis. An inverse correlation is found between the number of mitochondria (total count) and the decrease in MtMP, while a direct correlation between the ROS concentration and the number of mitochondrial objects is detected (*r*-Pearson coefficients are given in Table S1). The decrease in the total branch length, total branch length/mito, branch junctions, and branch junctions/mito has a direct correlation with MtMP and an inverse correlation with an increase in ROS. Thus, the changes in MtMP and ROS concentration are correlated with mitochondrial fragmentation. Moreover, a strong correlation is observed between the decreasing MtMP and increasing ROS concentration.

Table S1. Pearson correlation coefficients for changes in MtMP, ROS concentration, and mitochondrial network morphology parameters. Data are presented as *r*-Pearson, where $^{*}p < 0.05$ and $^{**}p < 0.001$ indicate statistically significant differences.

Parameter	Total Count	Total Branch Length	Total Branch Length/mito	Branch Junctions	Junctions/mito
**ROS**	0.67*	-0.95*	-0.88*	-0.85*	-0.87*
**MtMP**	-0.55**	0.93*	0.85*	0.85*	0.86*
